# Balancing Privacy Protection and Reproducible Entity Extraction in Real-World Data in the Development of a Framework for Deidentified Japanese Electronic Health Record Narratives Using BERT: Retrospective Observational Study

**DOI:** 10.2196/97411

**Published:** 2026-11-06

**Authors:** Nobuo Mochizuki, Atsushi Ikeda, Hideyuki Ohshima, Yukika Fukuda, Kyoko Okuda, Ryota Komaki, Tomoyuki Matsunaga, Kenichi Kudo, Shintaro Goto, Motoki Amai, Yusuke Suzuki

**Affiliations:** 1 Health Data Business Development Center, Business Development Division, TOPPAN Holdings Inc., 1-3-3, Suido, Bunkyo-ku, Tokyo, 112-8531, Japan, 81 3-3835-5111; 2 Japan Medical Association Medical Information Management Organization, Bunkyo-ku, Tokyo, Japan; 3 Department of Data Management, Integrated Clinical Care Informatics Inc, Bunkyo-ku, Tokyo, Japan

**Keywords:** data anonymization, electronic health records, hospitals, natural language processing, neoplasms, predictive value of tests, prescriptions, privacy

## Abstract

**Background:**

Pharmacoepidemiologic studies using real-world data often lack clinical context embedded in free-text electronic health records (EHRs). However, sharing EHR narratives is restricted by privacy requirements, and conventional deidentification approaches may reduce analytic utility and limit auditability.

**Objective:**

This study aims to develop and evaluate a framework that (1) extracts structured cancer-related entities from Japanese EHR free text using BERT (Bidirectional Encoder Representations from Transformers)–based natural language processing and (2) generates deidentified analytic text that preserves contextual information for post hoc auditing under privacy constraints.

**Methods:**

This retrospective observational study used the DATuM IDEA database, which integrates EHR narratives with structured records (claims, prescriptions, and procedure/surgery records) from 2 hospitals in Japan from January 1, 2019, through June 30, 2025. Deterministic linkage was performed within ICI (Integrated Clinical Care Informatics Inc) prior to deidentification. Downstream recoverability analyses were conducted within a governance-constrained analyzable text cohort derived after deidentification and preprocessing. We quantified deidentification using masking rates and evaluated downstream recoverability of TNM (tumor-node-metastasis) or stage-related mentions, internal logical consistency (M1 vs Stage IV), and proxy-based concordance using structured data treatment proxies (Anatomical Therapeutic Chemical [ATC] code L for systemic anticancer therapy and procedure-name keywords for cancer-directed interventions). A stratified manual audit of 400 deidentified narratives primarily inspected for apparent residual direct identifiers and secondarily for retention of TNM and stage outputs.

**Results:**

Among 10,214 patients with neoplasms (*International Statistical Classification of Diseases, Tenth Revision* [*ICD-10*] C00-D48), linked progress notes were available for 4383 patients (82,863 records). The analyzable text cohort (Layer C) comprised 3689 patients and 38,841 documents, of which 1339 patients (1339/3689, 36.3%) and 11,914 documents (11,914/38,841, 30.7%) had at least one recoverable TNM or stage mention (Layer D). Marginal document-level recoverability in Layer C was 24.2% for T, N, and M elements and 23.6% for stage. Conditional on Layer D, it was 78.8% for T, 78.7% for N, 78.8% for M, and 76.8% for stage. Median masking rates ranged from 9.9% to 15% across major cancer categories. M1 and Stage IV mention indicators showed an overall agreement of 94.7%, positive percent agreement of 54.8%, and negative percent agreement of approximately 99%. Proxy-based concordance analysis showed positive predictive values of 0.700 for Stage IV using the systemic therapy proxy and 0.031 for early-stage classification using the procedure proxy. Manual audit demonstrated retention of intended TNM/stage-related outputs after normalization and identified no apparent residual direct identifiers in the audited deidentified narratives.

**Conclusions:**

This framework generates deidentified Japanese EHR narratives that preserve contextual structure for auditing while supporting structured entity extraction, addressing the trade-off between privacy protection and reproducibility in real-world data research.

## Introduction

### Missing Clinical Context in Pharmacoepidemiologic Studies

In pharmacoepidemiologic research, understanding adverse event trajectories and the clinical intent underlying drug prescribing requires narrative context embedded in free-text electronic health records (EHRs) [1,2]. However, structured data alone (eg, diagnosis codes and medication orders) often fail to capture the reasons for clinical decisions or the causal relationships between events, leading to critical information gaps that limit analytical validity and interpretability [3-5]. Manual chart review remains the gold standard for addressing these gaps, but it is labor-intensive, difficult to scale to large datasets, and prone to extraction errors [6,7].

### Challenge: Privacy-Utility Trade-Off in Sharing Clinical Narratives

Unstructured EHR narratives are increasingly recognized as a valuable resource for enriching structured datasets with clinically meaningful context. However, sharing raw clinical text is constrained by stringent privacy requirements [2,5,6,8]. Conventional deidentification methods typically replace protected health information (PHI) with generic tags or remove it entirely. These approaches can obscure subtle yet clinically important expressions, resulting in a substantial loss of analytical utility and limiting the ability to audit and validate findings against the original narrative context [1,9]. This challenge is particularly pronounced in Japanese clinical text, which often includes mixed Japanese-English expressions, ambiguous negation, and orthographic variation, making context-preserving automated deidentification especially difficult [10,11].

### Objective and Significance: Generating Auditable Deidentified Analytic Text

This study aimed to develop a framework that simultaneously (1) extracts structured information from Japanese EHR free text using BERT (Bidirectional Encoder Representations from Transformers)–based natural language processing (NLP) and (2) generates deidentified analytic text under governance constraints that preserves sufficient context to support post hoc validation.

We implemented a multistep deidentification process that begins with fully masked text and selectively restores clinically relevant keywords while retaining selected contextual expressions relevant to downstream interpretation and essential medical terminology. This approach was designed to reduce direct identifier exposure while preserving clinically relevant contextual cues, enabling the assessment of interevent relationships and clinical reasoning that are difficult to infer from structured data alone.

Using an oncology follow-up setting, we demonstrated how the combined use of structured variables and context-preserving deidentified narratives can enhance the reliability and efficiency of pharmacoepidemiologic research.

## Methods

### Study Design, Setting, Data Sources, and Governance

This retrospective observational study used the DATuM IDEA database, which was developed by TOPPAN Holdings Inc using data provided by the Medical Information Management Organization of the Japan Medical Association (J-MIMO) and Integrated Clinical Care Informatics Inc (ICI). Both J-MIMO and ICI are business operators certified by the Cabinet Office under Japan’s Act on Anonymized Medical Data That Are Meant to Contribute to Research and Development in the Medical Field (Act No. 28 of 2017; commonly referred to as the Next-Generation Medical Infrastructure Act) [12], which authorizes certified operators to create and provide anonymized medical data for research and development purposes.

The DATuM IDEA database integrates routinely collected EHR data including unstructured clinical narratives (eg, discharge summaries and progress notes), with structured records such as claims, prescriptions, and procedure or surgery data from 2 oncology care hospitals in Japan that provide both inpatient and outpatient services. Hospital names are not disclosed because of data governance requirements. The study data covered the period from January 1, 2019, through June 30, 2025. Because the data were retrospectively collected, a time lag existed between data generation and availability for analysis.

Under the governance framework established by the Act, data are collected and made available for research through patient notification and an opt-out mechanism. Before researcher access, data collection, linkage, and deidentification are conducted under certified governance, and TOPPAN Holdings Inc standardizes the EHR and claims data and provides the DATuM IDEA research database for approved projects. This study used deidentified data made available for research in accordance with the applicable legal and governance framework in Japan.

### Ethical Considerations

This study was conducted in accordance with the Declaration of Helsinki and under Japan’s Next-Generation Medical Infrastructure Act. The research and development plan was reviewed and approved by the Processed Information Provision Review Committee of the J-MIMO (approval number: 2023‐2). Because the data were collected under a patient notification and opt-out framework and provided in deidentified form under certified governance, the requirement for individual informed consent was waived.

### Data Access and Security

Identifiable source data were retained and processed within ICI. Deterministic linkage and deidentification were completed before data release. Researchers at TOPPAN accessed only deidentified outputs (including deidentified analytic text) and had no access to direct identifiers.

### Data Linkage

Within ICI, deterministic linkage was performed before deidentification at each participating hospital using facility-specific patient chart identifiers to link unstructured EHR narratives with structured records (claims, prescriptions, and procedure or surgery data). Patient identifiers were facility-specific and were not harmonized across participating facilities; therefore, the same individual appearing at different facilities, if applicable, would be represented by separate database person identifiers. Structured record linkage was available for 4346 of 4383 patients in the linked text cohort (99.2%). Unlinked records were excluded from analyses requiring structured data concordance.

### Study Population

Eligible patients were those with at least one recorded diagnosis within *International Statistical Classification of Diseases, Tenth Revision* (*ICD-10*) codes C00-D48 (neoplasms) during the study period. Because cancer diagnoses may have occurred before the start of the data capture period, “first recorded diagnosis” was defined as the earliest observed cancer diagnosis within the available dataset; we note that, in the database, the recorded diagnosis date could precede the period during which structured data become fully observable, which was a specification of this database rather than a general property of EHR data (see the Observation Time Definition section). The linked text cohort that served as the source text population for the deidentification framework consisted of all eligible patients with at least one linked progress note. All downstream recoverability and proxy-based concordance analyses reported in this manuscript were conducted in the downstream analyzable text cohort (Layer C), defined as patients meeting all of the following: (1) included in the linked text cohort; (2) first recorded cancer diagnosis on or after January 1, 2019; (3) *ICD-10* level-3 category not corresponding to secondary or unspecified neoplasms; and (4) belonging to an *ICD-10* level-3 category in which at least 50 patients met these criteria. Recoverable TNM (tumor-node-metastasis) or stage-related mentions identified within this cohort were summarized separately as the downstream recoverable subset (Layer D). Cohort sizes derived under these criteria are reported in the Study Population and Analytic Flow section. Accordingly, downstream recoverability estimates were calculated within this analyzable cohort, with Layer C used for marginal recoverability and Layer D used for conditional recoverability. These estimates should not be interpreted as performance estimates for an unselected representative corpus of all linked progress notes.

### Sampling Strategy

Notes were not preselected on the basis of prior chart confirmation of TNM or stage documentation; the entity extraction pipeline was applied to all linked progress notes in the eligible patient cohort. The construction of the analytic cohort is acknowledged in interpretation: Reported recoverability metrics characterize the pipeline’s behavior within this analyzable subset rather than its marginal recovery probability over an unselected corpus. For proxy-based concordance analyses (see the Evaluation section), only cancer types with at least 50 eligible patients in the analytic cohort were included, and stratified analyses were performed based on *ICD-10* level-3 category.

### Observation Time Definition

#### Descriptive Analysis

For descriptive analyses, observation time was defined as the total observation period window available in the database for each patient, calculated from the observation period start date to the observation period end date. We did not use the interval from the first observed cancer diagnosis date to the end of observation, because in the database, the recorded diagnosis date could precede the period during which structured data (eg, prescriptions and procedures) became fully observable. This temporal asymmetry was a specification of the database rather than a general property of EHR data and would, if used as the observation time, lead to underrepresentation of treatment-related observation time at the individual patient level.

#### Missing and Unavailable Data (RECORD Compliance)

In accordance with RECORD (Reporting of Studies Conducted Using Observational Routinely Collected Health Data) guidelines [13], we report variable-level data availability within the downstream analyzable text cohort (Layer C: 3689 patients, 38,841 documents). Patient-level variables (age at first cancer diagnosis, sex, observation time) were fully available (0% unavailability for all 3 variables). For document-level extraction variables, structural nonobservation rates in Layer C were 75.8% (29,457/38,841 documents without recoverable mention) for the T element, 75.8% (29,459/38,841) for the N element, 75.8% (29,457/38,841) for the M element, and 76.4% (29,691/38,841) for the overall stage. These rates reflect structural absence in the deidentified analytic text rather than conventional missing data. For proxy-based concordance variables, 70.3% (2594/3689) of Layer C patients had no antineoplastic drug record (Anatomical Therapeutic Chemical [ATC] code L) at any database time point; 73.8% (2724/3689) had no cancer-directed procedure record. These absences reflect structural absence from the contributing hospitals’ claims records. Variable-level statistics are summarized in Table S1 in Multimedia Appendix 1.

### NLP Model for Information Extraction

#### BERT Model

We used a BERT-based supervised learning model pretrained on general Japanese corpora and Japanese clinical text and subsequently adapted within the operational upstream extraction workflow used during data generation. The upstream extraction model was designed for broad clinical entity identification and was not specifically optimized for TNM- or stage-focused extraction during the original data generation process. Information extraction from unstructured narratives was formulated as a named entity recognition (NER) task targeting a predefined set of clinical entities (eg, TNM classification elements, overall stage expressions, and other oncology entities) and PHI categories (eg, personal names, addresses, and institution names). Of the clinical entities identified by the model, this manuscript focuses on TNM classification elements and clinical/pathological stage (Stage I-IV) as the targets of the evaluation conducted on deidentified analytic text. Data partitioning for model development and evaluation was performed at the patient level so that records from the same patient were assigned to only one partition. Separate training, validation, and hold-out test partitions were used for model development, hyperparameter tuning, and final evaluation, respectively. Hyperparameter tuning and model selection were based on the validation partition, and final evaluation was conducted on the hold-out test partition. Detailed development and evaluation procedures are described in the following section.

#### Negation Handling

The BERT-based extraction model labels entity spans regardless of negation context. A postextraction negation filtering step was applied to exclude negated clinical entity mentions. Extracted entity spans were classified as negated if their surrounding text context contained one or more of the 23 Japanese negation patterns in the curated negation pattern list (Table S3 in Multimedia Appendix 1). Patterns covered grammatical forms for absent findings (eg, 認めない, 認めず), negative test results (eg, 陰性でした, 陰性所見), and excluded diagnoses (eg, 否定済みのため, 感染症否定). Negated entities were excluded from the downstream analytic entity set before delivery. Negation expressions themselves are typically not restored by the whitelist and remain masked as asterisks in the deidentified analytic text, preventing misleading false-positive readings.

#### Model Development and Internal Reference Evaluation

For internal development and evaluation of the entity extraction component, we used a manually reviewed source text reference set comprising 125 documents from 125 patients across 2 hospitals, with 1 document per patient to ensure patient-level separation.

Before annotation and model input, the text_tag_info field was standardized by replacing tab characters with 4 half-width spaces and replacing line breaks (CR, LF, and CRLF) with HTML break markers.

The model development set consisted of 100 documents sampled from 5 document types: progress records, progress notes, referral letters, discharge or transfer nursing summaries, and discharge summaries. For the model development set, 20 documents were sampled from each document type. Sampling was restricted to documents whose character length fell between the first and third quartiles within each document type in this initial internal evaluation.

An independent hold-out test set consisted of 25 additional documents from 25 patients, with 5 documents sampled from each of the same 5 document types. The hold-out test set was not used for model training nor tuning. Within the 100-document model development set, data were further split at the patient level into training and validation partitions in an 8:2 ratio, yielding 80 training documents and 20 validation documents.

Manual annotation was conducted independently by 2 reviewers for each document, and discrepancies were resolved by discussion to create the final reference annotations. In this internal evaluation, a predicted entity was counted as correct only when the extracted span fully covered the target expression and the assigned label matched the reference annotation. Interannotator agreement (IAA) was calculated by treating Person 1 annotations as the reference set and Person 2 annotations as the prediction set; strict span-level *F*_1_ was symmetric and unchanged when the roles were reversed. Entity-level recall, precision, and *F*_1_ score were calculated as follows: recall=number of correctly extracted entities/number of reference entities; precision=number of correctly extracted entities/number of predicted entities; and *F*_1_ score=2×recall× precision/(recall+precision). IAA was additionally summarized using Cohen kappa at the entity-label level. This internal evaluation was conducted on raw source text within ICI and should be distinguished from the study-level extraction analyses reported elsewhere in this manuscript, which were conducted on deidentified analytic text generated through the entity extraction pipeline and whitelist-based deidentification process.

### Deidentification and Generation of Deidentified Analytic Text

#### Definition of PHI

Within the certified governance framework under Japan’s Next-Generation Medical Infrastructure Act, identifiers in clinical free text were defined as information whose retention in the released analytic text could plausibly enable patient reidentification. The masking policy implemented in this study targeted direct identifiers (eg, personal names and patient IDs), contact and location information (eg, addresses and telephone numbers), provider or facility identifiers (eg, hospital and clinic names), and other contextual expressions that could increase reidentification risk. In this implementation, calendar dates were intentionally retained in the released analytic text because temporal structure was necessary for downstream pharmacoepidemiologic analyses, including treatment timing, sequencing of clinical events, and time-to-event reasoning. Accordingly, the operational masking policy targeted direct and contextual identifiers while preserving clinically relevant oncology content and calendar dates needed for downstream analysis and auditing.

#### Multistep Deidentification and Generation of Deidentified Analytic Text

To balance privacy protection with analytic utility, deidentification was conducted within ICI using a text generation framework based on predefined safety standards for the secure secondary use of clinical narratives. Conceptually, the framework follows a conservative release policy: The released analytic text contains only content deemed nonidentifying and necessary for downstream analysis, rather than relying exclusively on detecting and removing sensitive information from the source text.

The final output was a deidentified analytic text generated under governance constraints that preserved sufficient narrative context to enable auditing of extracted entities while reducing direct identifier exposure. Because the deidentification framework includes proprietary components and is the subject of a Japanese patent application, detailed implementation code, internal data structures, and exhaustive rule inventories are not disclosed. Instead, this study reports the conceptual design, governance context, and empirical characteristics relevant to assessing privacy protection and analytic utility. A conceptual workflow description is provided in the next section.

#### Conceptual Overview of the Deidentification and Extraction Pipeline

The framework comprises 2 stages performed by separate parties under certified governance: an upstream stage executed within ICI (with access to identifiable source notes) and a downstream stage executed within TOPPAN (with access only to deidentified outputs). Both the BERT extraction layer and the privacy protection layer operate within ICI; only the deidentified analytic text together with the corresponding non-PHI entity outputs leave ICI to TOPPAN.

In stage A (within ICI), BERT-based entity extraction is applied directly to the raw source notes, identifying clinical entities (eg, TNM classification elements and overall stage expressions), and PHI categories (eg, personal names, contact information, addresses, institution names, patient identifiers) in a single pass. A whitelist dictionary of safe expressions is also consulted; the dictionary is content-biased, so Japanese function words such as conjunctions and particles have a low probability of being restored, whereas Japanese adverbs are more likely to be restored when matched, and English function words such as particles (“up,” “on,” “down,” “out”) and adpositions (“in,” “under,” “towards,” “before”) may be restored when matched. The deidentified analytic text is then generated by first replacing every character in the source text with a single-byte asterisk to produce a fully masked baseline then selectively restoring (1) BERT-extracted entities not labeled as PHI and (2) whitelist-matched tokens; all other characters remain masked. In this implementation, calendar dates are retained in the released analytic text because temporal information is required for downstream pharmacoepidemiologic analyses. The internal entity-level evaluation against the manually reviewed source text reference set (see the Model Development and Internal Reference Evaluation section) was performed at the BERT extraction step within ICI on raw source text, before the deidentified analytic text was generated. The outputs delivered from ICI to TOPPAN are the deidentified analytic text and the corresponding non-PHI entity outputs.

In stage B (within TOPPAN), TOPPAN receives the deidentified analytic text and the upstream non-PHI entity outputs under the certified governance scheme. For the proxy-based concordance assessments reported in this manuscript, T, N, M, and stage tokens were re-extracted from the deidentified analytic text using SQL-based queries (independently of the upstream BERT-derived entity outputs) and were then aggregated at the patient level for comparison with structured data treatment proxies.

The masking policy implemented in stage A covers categories such as personal names, contact information, addresses, institution names, patient identifiers, and other direct or quasi-identifying expressions while retaining clinically relevant oncology content (disease site, histology, TNM classification, overall stage, biomarker information) required for downstream extraction and auditing. A schematic of the workflow with stepwise pseudocode is provided in the Conceptual Workflow for Deidentification and Entity Extraction section in Multimedia Appendix 2, and a fully synthetic side-by-side illustration of the transformation from source-style text to deidentified analytic text is shown in Table 1.

**Table 1.** Fully synthetic example illustrating transformation from source-style Japanese clinical text to deidentified analytic text. All content shown is fictitious and was created solely for illustration of the framework; it does not correspond to any real patient, institution, or clinical encounter.

Format	Predeidentification input	Deidentified analytic text
Japanese, fully synthetic^a^	2022年5月12日、 仮名 名称様(68歳女性)が東京中央病院 呼吸器内科を受診。右肺上葉腺癌と診断。臨床病期 cT2aN1M0、Stage IIB。^b^ EGFR 変異陽性。新宿匿名クリニックよりご紹介。次回2022年6月2日に再診予定。^c^	2022年5月12日、 *****様(68歳女性)*******^d^ 呼吸器内科*受診。右肺上葉腺癌*診断。臨床病期 cT2aN1M0、 Stage IIB。EGFR 変異陽性。***********ご紹介。次回2022年6月2日*再診予定。
English gloss^e^	On May 12, 2022, Ms Kamei Meisho (68-y-old woman) visited the Department of Respiratory Medicine at Tokyo Central Hospital. Diagnosed with adenocarcinoma of the right upper lobe of the lung. Clinical stage cT2aN1M0, Stage IIB. EGFR mutation positive. Referred from Shinjuku Anonymous Clinic. Follow-up scheduled on June 2, 2022.	On May 12, 2022, ************ (68-y-old woman) visited the Department of Respiratory Medicine ** ***** ****** ********. Diagnosed with adenocarcinoma of the right upper lobe of the lung. Clinical stage cT2aN1M0, Stage IIB. EGFR mutation positive. Referred from **********. Follow-up scheduled on June 2, 2022.^f^

a The framework operates on Japanese clinical text.

b Clinically relevant oncology information (disease site, histology, TNM (tumor-node-metastasis) classification, overall stage, and biomarker status) was retained.

c In accordance with the operational masking policy, calendar dates were intentionally retained to preserve temporal structure required for downstream pharmacoepidemiologic analyses.

d Direct and contextual identifiers (personal names, institution names) are masked using single-byte asterisks.

e English glosses are provided for international readers and do not represent system output.

f The numbers of asterisks in the English gloss are illustrative.

### Evaluation

#### Evaluation Framework

The extraction-related analyses reported in this study were conducted in the downstream analyzable text cohort (Layer C). Accordingly, the reported recoverability metrics, internal logical consistency measures, and proxy-based concordance analyses should be interpreted as cohort-specific estimates rather than as performance estimates from an unselected representative sample of all linked progress notes.

The evaluation framework was designed for a retrospective observational study conducted in a governed real-world data environment. It therefore combined operational recoverability metrics, internal consistency checks, proxy-based concordance with structured data, and a targeted manual audit of generated outputs.

#### Deidentification Metrics (Masking and Restoration Rates)

To quantify redaction volume, we calculated the text opacity rate (masking rate). This metric measures the proportion of source characters replaced by asterisks and does not constitute a measure of readability, linguistic coherence, or privacy guarantee. For reference, we also report residual character density (100% − opacity rate). The opacity rate was calculated as follows:

Opacity rate (%) = (number of masked characters / total number of characters) × 100

Masking was represented by a single-byte asterisk character (“*”). Character counts were computed in Snowflake SQL using the LENGTH() function applied to the full text. The number of masked characters was calculated as the difference between the total text length and the length after removing asterisks (ie, LENGTH(text) - LENGTH(REPLACE(text,‘*,’''))).

#### Downstream Recoverability of TNM and Stage-Related Mentions

In the study-level analyses of deidentified analytic text, recoverability was defined as generation of a normalized target output for the prespecified entity of interest (eg, a T, N, or M component or an overall stage label) according to the predefined extraction and normalization rules. The document-level rates reported in this study therefore describe the proportion of analyzed documents within the governance-constrained analyzable cohort in which each individual TNM element was recoverable and normalized. They should not be interpreted as marginal extraction probabilities over an unrestricted corpus. This study-level operational metric is distinct from the entity-level recall, precision, and *F*_1_ evaluation performed on the manually reviewed source text reference set described in the Model Development and Internal Reference Evaluation section.

For patients with more than one associated document, component-level recoverability (T, N, M, and overall stage mention) was defined using an any-positive-mention rule: A component was considered recoverable at the patient level if it was identified in at least one of the patient’s documents. In addition, for the subset of patients with an explicit numeric stage mention (Stage I–IV) in 2 or more documents, conflicting stage values across documents were reconciled by assigning the most advanced (highest) stage value observed across the patient’s document set as the patient-level stage estimate. Among the 958 patients with an explicit stage value in 2 or more documents, 7.8% (75/958) exhibited multistage profiles (conflicting stage values across documents), with a mean stage spread (maximum minus minimum observed stage value) of 0.14. These figures characterize the extent of within-patient conflicting extractions rather than asserting a single definitive clinical stage.

#### Internal Logical Consistency

Internal logical consistency was assessed by measuring concordance between the independently extracted TNM element “M1” (distant metastasis) and the overall stage classification “Stage IV” using overall agreement, positive percent agreement (PPA), negative percent agreement (NPA), and Cohen kappa. Jaccard coefficients were additionally calculated for corresponding T-element and stage indicators at the document and patient levels to summarize positive-set overlap. Because the M1/Stage IV contingency tables were dominated by concordant-negative classifications with sparse positive observations, Cohen kappa was interpreted cautiously. The reported internal consistency measures were overall agreement, PPA, NPA, Cohen kappa, and Jaccard coefficients. For descriptive purposes, we also report simple overall binary agreement between M1 and Stage IV presence/absence indicators.

#### Proxy-Based Concordance With Structured Data

To assess exploratory concordance between extracted stage mentions and observable treatment proxies, we compared the extracted stage with structured data treatment proxies within 60 days of the document update date.

Stage IV classifications were considered proxy-concordant when systemic anticancer therapy was observed. Systemic therapy was defined as any antineoplastic or immunomodulating agent (ATC code L) captured in inpatient and outpatient medication records, including injections and dispensed outpatient prescriptions. ATC codes were mapped using the DATuM IDEA implementation.

Stage I-II classifications were assessed in relation to cancer-directed procedures as a proxy for curative-intent treatment. Procedures were identified from Japanese claims data using an OR-based keyword search on procedure name strings (Procedure Keyword List for Identifying Cancer-Directed Procedures in Japanese Claims section in Multimedia Appendix 2). Because keyword-based identification may capture heterogeneous interventions and may not reflect care delivered outside contributing hospitals or outside the observation window, this proxy-based concordance analysis was interpreted as concordance with observable treatments rather than verification of the true pathologic stage.

### Incident and Prevalent Case Classification and Time Window Sensitivity

Of the 3689 patients in Layer C, 1556 (42.2%) were classified as incident (first recorded cancer diagnosis ≥30 days after the data capture start date for their hospital), 2016 (54.6%) were classified as prevalent (first diagnosis predating the data capture start date), and 117 (3.2%) were considered unclassifiable (0‐29 d from data capture start). Among incident patients (n=1556), the proportions with at least one ATC code L record within 30, 60, and 90 days of first diagnosis were 14.8% (230/1556), 21% (327/1556), and 24.3% (378/1556), respectively—stable across window specifications. Prevalent patients showed markedly lower rates (8/2016, 0.4%; 30/2016, 1.5%; and 62/2016, 3.1%, for 30, 60, and 90 d, respectively), consistent with treatment history predating the database observation window. All-patient rates across the same time windows were 6.8% (251/3689), 10.2% (376/3689), and 12.6% (465/3689), respectively. These results are summarized in Table S4 in Multimedia Appendix 1.

### Manual Audit (Sanity Check) and Residual PHI Inspection

A manual audit of the generated deidentified analytic text was conducted primarily to inspect for apparent residual direct identifiers. Retention of intended TNM classification and cancer stage outputs after normalization was reviewed as a secondary sanity check. The audit included a stratified sample of 400 deidentified narratives limited to cancer types with at least 50 verifiable cases, with allocation proportional to patient counts per cancer type. TNM classification and stage were manually reviewed within the audited generated outputs to assess whether the intended extracted entities were retained after normalization.

Given that the deidentified analytic text is generated to explicitly reflect entities extracted by the NLP pipeline, this audit served as a sanity check for entity retention and residual PHI inspection rather than as a formal gold standard evaluation of general NLP performance (eg, precision, recall, and *F*_1_) on raw clinical narratives. For operational data release, a multilayer quality assurance process is planned, combining sample-based manual review with automated PHI screening using SQL-based pattern checks.

## Results

### Data Linkage

Deterministic linkage was performed within ICI before deidentification using facility-specific patient identifiers. Among the 4383 patients in the linked text cohort, structured-record linkage was available for 4346 patients (99.2%) for downstream proxy-based concordance analyses.

### Internal Reference Evaluation of the Extraction Model

Two independent annotators each annotated the same document set for target clinical entities (Person 1: 861 entities; Person 2: 904 entities). Strict span-level IAA, requiring agreement on both span boundaries and entity label, achieved an *F*_1_ of 0.593 (precision: 0.579, recall: 0.607), with Person 1 treated as the reference set and Person 2 treated as the prediction set for the reported calculation; the value was symmetric and unchanged when the roles were reversed. Cohen kappa at the entity-label level was 0.766, indicating substantial agreement. After joint reconciliation, the gold standard reference set contained 955 entities. Against this gold standard, the pretraining baseline achieved an *F*_1_ of 0.384 (precision: 0.329, recall: 0.461; 1339 predicted entities). The fine-tuned model achieved an *F*_1_ of 0.557 (precision: 0.552, recall: 0.561; 971 predicted entities), an absolute *F*_1_ improvement of +0.173 (+45% relative), and 27% reduction in over-prediction. The fine-tuned model *F*_1_ of 0.557 fell within 0.036 points of the IAA ceiling of 0.593 (Table S2 in Multimedia Appendix 1).

These results provided an initial internal source text reference evaluation of the extraction component and were distinct from the study-level recoverability analyses reported below for the governance-constrained analyzable cohort based on deidentified analytic text.

### Study Population and Analytic Flow

From the DATuM IDEA database, 51,876 patients had at least one recorded diagnosis. Among them, 10,214 patients had a diagnosis of neoplasms (*ICD-10* C00-D48) recorded on or after January 1, 2019. Linked progress notes were available for 4383 patients (82,863 records); this linked text cohort served as the source text population for the deidentification framework. Application of the downstream processing criteria described in the Study Population section yielded a downstream analyzable text cohort (Layer C) of 3689 patients and 38,841 documents that was used for downstream recoverability and proxy-based concordance analyses. Of these, 1339 patients (Layer D, 36.3% marginal patient-level recoverability) and 11,914 documents (30.7% marginal document-level recoverability) had at least one recoverable TNM or stage mention. See Figure 1 for the updated cohort attrition diagram. Accordingly, downstream recoverability estimates were interpreted within the governance-constrained Layer C cohort; estimates reported specifically for Layer D were interpreted as conditional on the presence of at least one recoverable TNM or stage mention.

**Figure 1.**
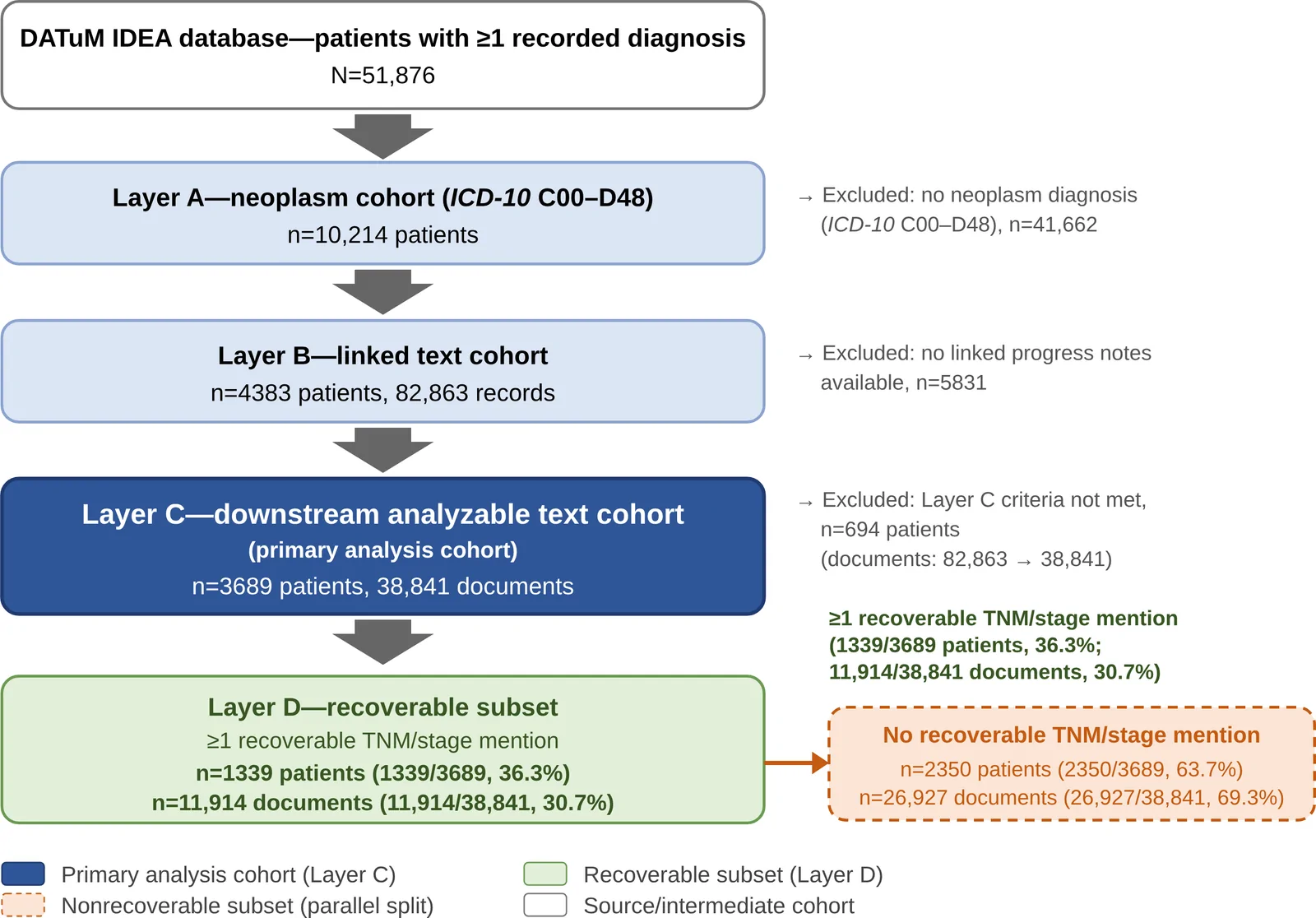
Study population and analytic flow for cohort identification, progress note availability, and the restricted extraction-focused evaluation subset. Layer C was the primary analysis cohort used for all extraction-related analyses. The orange dashed box is a parallel split of Layer C (not an excluded group). Source: DATuM IDEA database (linked hospital claims and progress note records). *ICD-10*: *International Statistical Classification of Diseases, Tenth Revision*; TNM: tumor-node-metastasis.

The flow diagram summarizes patients with at least one recorded diagnosis in DATuM IDEA (n=51,876), patients with neoplasms (*ICD-10* C00-D48; n=10,214), patients in the linked text cohort with available linked progress notes (n=4383; n=82,863 records), and the downstream analyzable text cohort (Layer C) used for downstream recoverability and proxy-based concordance analyses (Layer C: 3689 patients and 38,841 documents; Layer D: 1339 patients and 11,914 documents). Deterministic linkage between unstructured EHR narratives and structured records was performed within ICI prior to deidentification, with structured record linkage available for 4346 of 4383 patients (99.2%). Hospital names were not disclosed.

### Patient Characteristics and Observation Time in Major Cancer Subgroups

Patient and document characteristics for 6 major *ICD-10* level-3 cancer subgroups within the downstream analyzable text cohort (Layer C) are summarized in Table 2.

**Table 2.** Patient and document characteristics by *International Statistical Classification of Diseases, Tenth Revision* (*ICD-10*) level-3 cancer subgroup within the downstream analyzable text cohort (Layer C: n=3689 patients and 38,841 documents).

*ICD-10* code	Cancer subgroup^a^	Patients^b^, n	Documents^c^, n	Age at first observed cancer diagnosis (years)			Observation time^d^, (days)		
				Q1^e^	Median	Q3^f^	Q1	Median	Q3
C16	Malignant neoplasm of the stomach	214	1673	70	76	80	181	272	339
C18	Malignant neoplasm of the colon	200	1447	65	74	78	189	285	346
C20	Malignant neoplasm of the rectum	206	2337	60	70	76	197	297	345
C34	Malignant neoplasm of the bronchus and lung	248	2673	68	75	80	135	277.5	317
C50	Malignant neoplasm of the breast	265	2902	53	67	75	76.3	244.5	315
C61	Malignant neoplasm of the prostate	296	1994	71	75	79	176.5	252	321

a Defined by *ICD-10* level-3 codes within the downstream analyzable text cohort that had at least 50 patients meeting the analytic cohort criteria for subgroup reporting.

b Not mutually exclusive because patients could have neoplasms represented in more than one *ICD-10* level-3 category; therefore, the total could exceed the 3689 patients in the downstream analyzable text cohort (Layer C).

c Analyzed documents contributing to subgroup-specific extraction and proxy-based concordance analyses.

d Total observation period within the database for each patient.

e Q1: quartile 1.

f Q3: quartile 3.

### Downstream Recoverability, Internal Logical Consistency, and Proxy-Based Concordance

We evaluated TNM recoverability and assessed both internal consistency and concordance with structured data within the downstream analyzable text cohort (Layer C: 3689 patients, 38,841 documents). Within the Layer D subset (11,914 documents), the document-level recoverability rates for TNM elements were 0.788 for T, 0.787 for N, and 0.788 for M. These rates reflect the proportion of analyzed documents in which each element was recoverable and normalized within the governance-constrained analyzable cohort, corresponding to Layer C document-level marginal recoverability of 24.2% for T, N, and M elements and 23.6% for stage (see the Panel A information in Table 3 as well as Panel B information in Table 3 for M1/Stage IV concordance metrics). Internal concordance between independently extracted M1 and Stage IV mention indicators showed an overall agreement of 94.7%, a PPA of 54.8%, an NPA of approximately 99.0%, and a kappa near zero because of structural imbalance. Separately, the Jaccard coefficients for T and stage were 0.080 and 0.096, respectively, at the document level and 0.169 and 0.168, respectively, at the patient level.

In the structured data proxy analysis, Stage IV classification using systemic anticancer therapy (ATC code L) showed a sensitivity of 0.257, specificity of 0.822, and positive predictive value (PPV) of 0.700. Early-stage classification using cancer-directed procedures as a proxy for curative-intent treatment showed a sensitivity of 0.549, specificity of 0.582, and PPV of 0.031. Notably, cancer-specific PPVs for Stage IV were 0.800 (breast cancer), 0.800 (prostate cancer), 0.749 (lung cancer), 0.526 (rectal cancer), 0.456 (colon cancer), and 0.798 (gastric cancer); corresponding PPVs for early-stage classification were 0.023, 0.026, 0.008, 0.037, 0.065, and 0.035, respectively (see the Panel D information in Table 3).

**Table 3.** Downstream recoverability, internal logical consistency, and proxy-based concordance metrics.

Measure	Definition/proxy^a^	Estimate	Notes
Panel A. Marginal and conditional TNM^b^/stage recoverability rates			
T recoverability rate	Proportion of Layer C documents with a successfully extracted and normalized T element; the Layer D conditional rate reported separately	24.2% (Layer C marginal); 78.8% (Layer D conditional)	Layer C marginal rate: 9384/38,841 (24.2%) documents; Layer D conditional rate: 9384/11,914 (78.8%) documents; normalization applied.
N recoverability rate	Proportion of Layer C documents with a successfully extracted and normalized N element; the Layer D conditional rate reported separately	24.2% (Layer C marginal); 78.7% (Layer D conditional)	Layer C marginal rate: 9382/38,841 (24.2%) documents; Layer D conditional rate: 9382/11,914 (78.7%) documents; normalization applied
M recoverability rate	Proportion of Layer C documents with a successfully extracted and normalized M element; the Layer D conditional rate is reported separately.	24.2% (Layer C marginal); 78.8% (Layer D conditional)	Layer C marginal rate: 9384/38,841 (24.2%) documents; Layer D conditional rate: 9384/11,914 (78.8%) documents; normalization applied
Stage recoverability	Proportion of Layer C documents with a successfully extracted and normalized overall stage expression; the Layer D conditional rate reported separately	23.6% (Layer C marginal); 76.8% (Layer D conditional)	Layer C marginal rate: 9150/38,841 (23.6%) documents; Layer D conditional rate: 9150/11,914 (76.8%) documents; normalization applied
Panel B. Internal logical consistency			
Overall agreement, M1 vs Stage IV	Proportion of documents with concordant presence or absence of independently extracted M1 and Stage IV mentions	94.7%	Document-level binary concordance between M1 and Stage IV mention indicators; overall agreement, PPA^c^, and NPA^d^ reported as agreement-oriented measures; Cohen kappa near zero in the setting of low Stage IV prevalence, asymmetric positive rates, and a dominant concordant-negative cell
PPA, M1 vs Stage IV	Among documents with a Stage IV mention, the proportion that also contained an M1 mention	54.8%	Document-level PPA using Stage IV presence as the comparison-positive category; concordance between clinically related mention categories described by this measure, which does not represent diagnostic accuracy or validation against a clinical gold standard
NPA, M1 vs Stage IV	Among documents without a Stage IV mention, the proportion that also lacked an M1 mention	approximately 99%	Document-level negative percent agreement using Stage IV absence as the comparison-negative category; the high value is influenced by the large number of concordant-negative documents and should be interpreted as an agreement-oriented descriptive measure.
Document-level Jaccard coefficient, T	Overlap between the corresponding positive T-element indicators at the document level	0.080	Document-level positive-set overlap; concordant-negative documents are not included in the Jaccard calculation.
Document-level Jaccard coefficient, Stage	Overlap between the corresponding positive stage indicators at the document level	0.096	Document-level positive-set overlap; this metric is distinct from the M1 versus Stage IV concordance analysis.
Patient-level Jaccard coefficient, T	Overlap between patient-level positive T-element indicators aggregated using an any-positive-mention rule	0.169	Patient-level aggregation from document-level flags; concordant-negative patients are not included in the Jaccard calculation.
Patient-level Jaccard coefficient, Stage	Overlap between patient-level positive stage indicators aggregated using an any-positive-mention rule	0.168	Patient-level aggregation from document-level flags; this metric is distinct from the M1 versus Stage IV concordance analysis.
Panel C. Proxy-based concordance with structured data			
Stage IV (systemic therapy proxy)–sensitivity	Proportion of Stage IV classifications accompanied by systemic anticancer therapy within 60 days, using ATC code L as an observable treatment proxy	0.257	ATC^e^ mapping implemented within DATuM IDEA; inpatient/outpatient, injected drugs, and dispensed outpatient prescriptions included
Stage IV (systemic therapy proxy)–specificity	Proportion of non-Stage IV classifications not accompanied by the systemic therapy proxy within 60 days	0.822	Same proxy and time window as above (ATC code L; 60 days)
Stage IV (systemic therapy proxy)–PPV^f^	Proportion of classifications accompanied by the systemic therapy proxy that were classified as Stage IV	0.700	Exploratory proxy-based measure
Stage I-II^g^ (procedure proxy)–sensitivity	Cancer-directed procedures within 60 days identified via an OR-based keyword search of Japanese claims procedure-name strings	0.549	Procedures as proxy for curative-intent treatment; keyword list in the Procedure Keyword List for Identifying Cancer-Directed Procedures in Japanese Claims section in Multimedia Appendix 2
Stage I-II^g^ (procedure proxy)–specificity	Same as above	0.582	Same proxy and time window as above (cancer-directed procedures; 60 days)
Stage I-II^g^ (procedure proxy)–PPV^f^	Same as above	0.031	Exploratory proxy-based measure
Panel D. Cancer-specific proxy-based concordance (Stage IV systemic therapy proxy)			
Stage IV PPV^f^ gastric cancer (C16)	Systemic anticancer therapy proxy (ATC code L), same as Panel C	0.798	Early-stage PPV (procedure proxy): 0.035
Stage IV PPV^f^ colon cancer (C18)	Systemic anticancer therapy proxy (ATC code L), same as Panel C	0.456	Early-stage PPV (procedure proxy): 0.065
Stage IV PPV^f^ rectal cancer (C20)	Systemic anticancer therapy proxy (ATC code L), same as Panel C	0.526	Early-stage PPV (procedure proxy): 0.037
Stage IV PPV^f^ lung cancer (C34)	Systemic anticancer therapy proxy (ATC code L), same as Panel C	0.749	Early-stage PPV (procedure proxy): 0.008
Stage IV PPV^f^ breast cancer (C50)	Systemic anticancer therapy proxy (ATC code L), same as Panel C	0.800	Early-stage PPV (procedure proxy): 0.023
Stage IV PPV^f^ prostate cancer (C61)	Systemic anticancer therapy proxy (ATC code L), same as Panel C	0.800	Early-stage PPV (procedure proxy): 0.026

a Proxy-based concordance analyses used structured-data treatment proxies observed within 60 days of the document update date.

b TNM: tumor-node-metastasis.

c PPA: positive percent agreement.

d NPA: negative percent agreement.

e ATC: Anatomical Therapeutic Chemical.

f Positive predictive value (PPV) estimates, including cancer-specific PPVs, were calculated with respect to the specified structured data treatment proxy and are reported as exploratory proxy-based measures. They should not be interpreted as diagnostic accuracy or agreement with a clinical gold standard.

g Stage I–II proxy-based concordance used cancer-directed procedures as an exploratory proxy for curative-intent treatment. Procedures were identified using an OR-based keyword search of Japanese claims procedure-name strings (the Procedure Keyword List for Identifying Cancer-Directed Procedures in Japanese Claims section in Multimedia Appendix 2).

### Deidentification Metrics by *ICD-10* Category

We evaluated the deidentification process by reporting masking and restoration rates across *ICD-10* categories to characterize the privacy-utility balance of the generated analytic texts. Deidentification metrics stratified by *ICD-10* category are summarized in Table 4.

The median masking rate ranged from 9.9% (C20, rectal cancer) to 15% (C34, lung cancer). The upper quartile (Q3) masking rate was highest for prostate cancer (C61; 21.3%). Corresponding restoration rates (100 − masking rate) are provided as a descriptive index of residual visible character density.

**Table 4.** Deidentification metrics, including masking and restoration rates, by *International Statistical Classification of Diseases, Tenth Revision* (*ICD-10*) category in the downstream analyzable text cohort (Layer C).

*ICD-10* code	Cancer subgroup	Documents, n^a^	Masking^b^ rate (%)			Restoration^c^ rate (%)		
			Q1^d^	Median	Q3^e^	Q1	Median	Q3
C16	Malignant neoplasm of the stomach	1673	7.8	11.0	14.8	85.2	89.0	92.2
C18	Malignant neoplasm of the colon	1447	7.1	10.7	14.9	85.1	89.3	92.9
C20	Malignant neoplasm of the rectum	2337	7.1	9.9	14.1	85.9	90.1	92.9
C34	Malignant neoplasm of the bronchus and lung	2673	11.1	15.0	20.0	80.0	85.0	88.9
C50	Malignant neoplasm of the breast	2902	8.8	11.8	15.5	84.5	88.2	91.2
C61	Malignant neoplasm of the prostate	1994	9.3	14.6	21.3	78.7	85.4	90.7

a Patients with neoplasms in more than one *ICD-10* level-3 category contribute to each applicable subgroup row, and the row-level document counts in this table reflect the same convention as in Table 2.

b Represented by the asterisk character (“*”): masking rate (%) = masked characters/total characters × 100.

c Defined as 100 − masking rate.

d Q1: quartile 1.

e Q3: quartile 3.

### Manual Audit (Sanity Check) and PHI Inspection

We also conducted a targeted manual audit of the generated deidentified analytic text using a stratified sample of 400 narratives. Among audited documents containing an intended TNM or stage output, all prespecified outputs were retained after normalization, and no apparent residual direct identifiers, including personal names, initials, facility names, addresses, telephone numbers, and patient IDs, were identified in any of the 400 audited narratives. Because the deidentified analytic text explicitly reflects entities generated through the extraction pipeline, this audit should be interpreted as a sanity check for output retention and PHI inspection rather than as a formal benchmark evaluation of precision, recall, or *F*_1_ on raw clinical narratives.

## Discussion

### Principal Findings

We developed a framework that provides both (1) structured variables extracted from Japanese EHR free text (eg, TNM elements and cancer stage) and (2) context-preserving deidentified analytic text to enable auditing of extracted outputs. In DATuM IDEA, 10,214 patients had a diagnosis of neoplasms (*ICD-10* C00-D48), and linked progress notes were available for 4383 patients (82,863 records); this linked text cohort defined the source text population for the deidentification framework. All downstream recoverability and proxy-based concordance analyses were conducted in the downstream analyzable text cohort (Layer C: 3689 patients and 38,841 documents), defined by the criteria described in the Study Population section. Deterministic linkage was performed within ICI before deidentification, and structured-record linkage was available for 4346 of 4383 patients (99.2%) in the linked text cohort.

### Balancing Privacy Protection and Analytic Utility

Our framework was designed to reduce reidentification risk while preserving narrative structure, including clinically meaningful terms, function words, and contextual cues. Previous work has addressed deidentification of Japanese EHR free text [9], whereas our framework further emphasizes the provision of deidentified analytic text that remains useful for auditing and reproducible extraction in studies using real-world data. This approach is particularly valuable in studies using real-world data, where structured data alone may not capture clinical intent, negation, uncertainty, or temporal context. Prior work has also shown that deidentification may affect downstream clinical NLP performance, underscoring the practical importance of balancing privacy protection against analytic utility rather than treating masking as a purely one-way process [10]. Providing auditable deidentified analytic text alongside structured variables offers a practical alternative to full manual chart review under privacy constraints.

### Extraction/Structuring Quality and Error Characteristics

Within the Layer D subset of 11,914 documents, the conditional document-level recoverability rates were 0.788 for T, 0.787 for N, and 0.788 for M. In contrast, across all Layer C documents, the marginal document-level recoverability rate was 24.2% for each of the T, N, and M elements and 23.6% for stage. The independently extracted M1 and Stage IV mention indicators showed high overall agreement, with an overall agreement of 94.7%, a PPA of 54.8%, and an NPA of approximately 99%. Cohen kappa remained near zero because of low positive prevalence, asymmetric positive rates, and a dominant concordant-negative cell. The TNM recoverability estimates should be interpreted in relation to the aforementioned Layer C and Layer D denominators and not as benchmark performance estimates from an unselected corpus of all oncology notes.

### Interpretation of Proxy-Based Concordance: Low PPV for Early-Stage Disease

In the structured data proxy analysis, Stage IV classification assessed against systemic anticancer therapy (ATC code L) showed a PPV of 0.700, supporting the plausibility of advanced/metastatic classification when an observable treatment proxy is available. In contrast, early-stage disease (Stage I–II) assessed in relation to cancer-directed procedures as a proxy for curative-intent treatment exhibited a low PPV (0.031).

This finding should not be interpreted as definitive evidence of incorrect stage assignment by the NLP component. Rather, it likely reflects limitations of using procedure-based proxies in routinely collected structured data. In particular, (1) early-stage cancer management is not uniformly surgical (eg, endoscopic therapy, radiotherapy, or active surveillance), (2) procedures may not be observed if care is delivered outside the contributing hospitals or outside the observation window, (3) timing misalignment can occur between narrative documentation of stage and billing-based capture of procedures, and (4) stage statements in notes may sometimes refer to prior or concurrent malignancies rather than the indexed primary cancer. More broadly, the use of structured treatment data as a proxy-based concordance strategy is consistent with a line of EHR research in which information extracted from narrative records is evaluated through downstream clinical consistency and treatment-related variables rather than through a single universal gold standard [11]. Accordingly, proxy-based concordance for early-stage disease should be interpreted as concordance with an observable treatment proxy within the available data window, rather than as verification of the true underlying pathologic stage. Future work will refine proxy-based concordance analyses using composite treatment proxies—including endoscopic therapy and radiotherapy—to further assess the robustness of the classification framework.

### Manual Audit of Deidentified Outputs

A targeted manual audit was performed on a stratified sample of 400 deidentified narratives from cancer types with at least 50 verifiable cases. Two independent auditors reviewed the narratives, including approximately 30 overlapping documents for inter-reviewer consistency. The audit focused on inspecting the deidentified outputs for apparent residual direct identifiers, including personal names, initials, facility names, addresses, telephone numbers, and patient IDs.

Because the analytic text explicitly reflects entities extracted by the NLP pipeline, this audit served as a sanity check of the generated output rather than a measure of general-purpose NLP performance on raw narratives. No apparent residual direct identifiers were detected in the audited sample; however, this does not exclude the possibility of residual direct identifiers outside the sample. For operational data release, we plan to implement a multilayer quality assurance process combining sample-based manual review with automated PHI screening using SQL-based pattern checks.

### Limitations and Future Directions

This study has several limitations. First, although the source text population consisted of all available linked progress note records in the eligible cancer cohort, conditional recoverability estimates (Layer D) are restricted to documents with at least one recoverable mention and would overstate recoverability; therefore, we also reported marginal extraction recoverability (document level: 30.7%; patient level: 36.3%) across the full downstream analyzable text cohort (Layer C). Accordingly, the reported recoverability metrics should not be interpreted as unbiased performance estimates for an entirely unselected corpus of all oncology progress notes.

Second, the internal reference evaluation annotation corpus was restricted to documents with character lengths between the first and third quartiles (Q1-Q3) within each document type to keep the annotation tractable within the governance-constrained budget. Entity-level metrics may not generalize to extreme-length documents.

Third, *ICD-10* level-3 cancer categories with fewer than 50 patients were excluded from subgroup analyses (Table 2) to ensure stable estimates. Performance on rare cancer categories cannot be inferred from the present results.

Fourth, the NLP annotation corpus was small (100-document model-development set; 25-document hold-out). No comparison against rule-based systems nor off-the-shelf clinical NER benchmarks was performed; the IAA *F*_1_ of 0.593 provides the primary human performance contextualization of the fine-tuned model *F*_1_ of 0.557.

Fifth, the ATC code L Stage IV proxy includes neoadjuvant and adjuvant therapies for early- to intermediate-stage disease. The concordance analysis was exploratory. The early-stage procedure proxy had a very low PPV (0.031) due to an inadequate keyword list; it was retained as illustrative only.

Sixth, short median observation times (244.5‐297 d for major cancer subgroups) constrained the proxy-based concordance analysis. The time window sensitivity analyses (30/60/90 d) and incident/prevalent stratification characterized this effect.

Seventh, the masking rate (text opacity rate) quantified redaction volume and did not constitute a privacy guarantee. Calendar dates were retained by design; quasi-identifier risk for rare cancer patients was acknowledged. Risk management relied on governance-level access controls. Formal readability assessment of the deidentified analytic text was not performed.

Eighth, this study used data from 2 hospitals, which remain unnamed due to governance requirements, and generalizability across institutions and documentation styles has yet to be established. Generalizability remains an important consideration for Japanese clinical NLP, as prior work has highlighted the challenges of cross-hospital deployment even for well-defined extraction tasks [8,11]. Scaling to 50 or more sites will require continuous monitoring for distribution shift, site-stratified performance assessment, and governance frameworks for model updates and PHI quality assurance.

Ninth, because the production implementation includes proprietary components and was executed within a certified governance environment, executable code and real example data cannot be released publicly. To improve transparency as far as feasible, we provide a conceptual workflow description, masking policy categories, stepwise pseudocode (Conceptual Workflow for Deidentification and Entity Extraction section in Multimedia Appendix 2), and a synthetic side-by-side example illustrating how clinically relevant oncology information is retained in deidentified analytic text (Table 1).

Tenth, detailed corpus description statistics for the annotation partitions (total character count; average document length; document type distribution; and PHI entity class distribution across categories such as personal name, date, institution name, patient ID, address, and contact information) were not computed in this study. Such analysis requires additional processing of raw source text within ICI, which retains exclusive access to identifiable source notes and PHI-labeled entities under the governance framework separating upstream and downstream processing; this was outside the scope of this study and is noted as an opportunity for future methodological transparency.

Eleventh, the population selection algorithms defining the cancer cohort (*ICD-10* C00–D48) and the downstream analyzable text cohort (Layer C 5-criteria inclusion rule) have not been independently validated against a patient-level gold standard or external electronic case report form review. Validation of these population selection criteria is acknowledged as a priority for future work when governance arrangements permit.

### Conclusions

We developed a framework that delivers deidentified Japanese EHR narratives while preserving narrative structure for auditing alongside structured entity outputs. This approach addresses the privacy-reproducibility trade-off inherent in real-world data research. With high-yield linkage between narratives and structured records and cohort-specific evidence of feasible extraction and deidentification, the framework has the potential to enhance the efficiency and credibility of pharmacoepidemiologic analyses leveraging Japanese EHR free text and claims data.

## Supplementary material

10.2196/97411 Multimedia Appendix 1 Variable availability for analysis variables in the downstream analyzable text cohort, inter-annotator agreement and natural language processing (NLP) model performance against gold standard reference set, Japanese negation patterns used in postextraction negation filtering, and incidence and prevalence classification and time-window sensitivity analysis for ATC code L records.

10.2196/97411 Multimedia Appendix 2 Procedure keyword list for proxy-based concordance analyses and conceptual workflow for governance-constrained deidentification and entity extraction.

10.2196/97411 Checklist 1 The RECORD statement checklist.

## Abbreviations

BERT: Bidirectional Encoder Representations from Transformers
EHR: electronic health record
IAA: interannotator agreement
*ICD-10*: *International Statistical Classification of Diseases, Tenth Revision*
ICI: Integrated Clinical Care Informatics Inc
J-MIMO: Medical Information Management Organization of the Japan Medical Association
NER: named entity recognition
NLP: natural language processing
NPA: negative percent agreement
PHI: protected health information
PPA: positive percent agreement
PPV: positive predictive value
RECORD: Reporting of Studies Conducted Using Observational Routinely Collected Health Data
TNM: tumor-node-metastasis
