# Supplementary material for: Balancing Privacy Protection and Reproducible Entity Extraction in Real-World Data in the Development of a Framework for Deidentified Japanese Electronic Health Record Narratives Using BERT: Retrospective Observational Study
Source: JMIR Med Inform. 2026 Nov 6;14:e97411. doi: 10.2196/97411 (PMC13743013; doi:10.2196/97411)
Supplement: Supplementary file 1 [file medinform-v14-e97411-s001.docx]

**Table S1.** Variable Availability for Analysis Variables in the Downstream Analyzable Text Cohort (Layer C) — RECORD Compliance

| **Variable** | **Level** | **N (denominator)** | **N Available** | **N Unavailable** | **% Unavailable** |
| --- | --- | --- | --- | --- | --- |
| **Patient-level variables (Layer C: n = 3,689 patients)** | | | | | |
| Age at first cancer diagnosis | Patient | 3,689 | 3,689 | 0 | 0.0% |
| Sex | Patient | 3,689 | 3,689 | 0 | 0.0% |
| Observation time (data availability window) | Patient | 3,689 | 3,689 | 0 | 0.0% |
| **Document-level extraction variables (Layer C: n = 38,841 documents)** | | | | | |
| T-element mention recoverable | Document | 38,841 | 9,384 | 29,457 | 75.8% |
| N-element mention recoverable | Document | 38,841 | 9,382 | 29,459 | 75.8% |
| M-element mention recoverable | Document | 38,841 | 9,384 | 29,457 | 75.8% |
| Overall stage mention recoverable | Document | 38,841 | 9,150 | 29,691 | 76.4% |
| **Proxy-validation variables (Layer C: n = 3,689 patients)** | | | | | |
| Systemic antineoplastic therapy record (ATC code L, any time) | Patient | 3,689 | 1,095 | 2,594 | 70.3% |
| Cancer-directed procedure record (keyword-based, any time) | Patient | 3,689 | 965 | 2,724 | 73.8% |

*Notes: "N Available" = documents or patients with a recoverable value. Structural absence indicates that the field was not present in the deidentified analytic text (for extraction variables) or in the contributing hospitals' claims records (for proxy-validation variables), and is distinct from conventional missing-at-random data. ATC = Anatomical Therapeutic Chemical classification.*

**Table S2.** Inter-Annotator Agreement and NLP Model Performance Against Gold-Standard Reference Set

| **Comparison** | **Reference entities (N)** | **Predicted entities (N)** | **TP** | **Precision** | **Recall** | **F1** | **Cohen's κ** |
| --- | --- | --- | --- | --- | --- | --- | --- |
| IAA: Person 1 vs Person 2 | 861 / 904 | 904 | 523 | 0.579 | 0.607 | **0.593** | 0.766 |
| Pre-training model vs gold-standard | 955 | 1,339 | 440 | 0.329 | 0.461 | **0.384** | — |
| Fine-tuned model vs gold-standard | 955 | 971 | 536 | 0.552 | 0.561 | **0.557** | — |

*Notes: IAA = inter-annotator agreement (strict span-level, requiring agreement on both span boundaries and entity label). TP = true positives (matching spans). Precision = TP / predicted entities. Recall = TP / reference entities. F1 = harmonic mean of precision and recall. Cohen's κ reported at entity-label level for IAA only; not applicable (—) for model evaluations due to structural class imbalance. Gold-standard reference set was created by joint reconciliation of Person 1 and Person 2 annotations (955 total entities). Pre-training model used a general Japanese BERT checkpoint without task-specific fine-tuning.*

**Table S3.** Japanese Negation Patterns Used in Post-Extraction Negation Filtering (n = 23 Patterns)

| **No.** | **Pattern (Japanese)** | **Category** | **Example context** |
| --- | --- | --- | --- |
| 1 | 認めない | Grammatical negation (non-past) | リンパ節転移を認めない |
| 2 | 認めず | Grammatical negation (conjunctive) | 進行を認めず |
| 3 | 認めなかった | Grammatical negation (past) | N2転移を認めなかった |
| 4 | 見られない | Absence observation (non-past) | 遠隔転移は見られない |
| 5 | 見られず | Absence observation (conjunctive) | 進行は見られず |
| 6 | 見られなかった | Absence observation (past) | 転移巣は見られなかった |
| 7 | なし | Absence noun | リンパ節転移なし |
| 8 | ない | Absence adjective (non-past) | 転移はない |
| 9 | なかった | Absence adjective (past) | Stage IVの根拠はなかった |
| 10 | 陰性 | Negative result | センチネルリンパ節陰性 |
| 11 | 陰性でした | Negative result (past polite) | 生検の病理所見は陰性でした |
| 12 | 陰性所見 | Negative finding noun | 画像上に陰性所見 |
| 13 | 陰性と判断 | Negative judgment expression | 病理所見は陰性と判断 |
| 14 | 陰性と考えられ | Negative inference | リンパ節は陰性と考えられ |
| 15 | 否定 | Rule-out (noun) | 遠隔転移は否定 |
| 16 | 否定された | Rule-out (past passive) | 他部位への転移は否定された |
| 17 | 否定済み | Already excluded | 感染症否定済みのため |
| 18 | 否定的 | Negative tendency | 転移は否定的 |
| 19 | 否定のため | Because excluded | 転移否定のため加療不要 |
| 20 | 除外 | Excluded (clinical) | ステージIVは除外 |
| 21 | 除外した | Was excluded (past) | 他の鑑別診断を除外した |
| 22 | 不検出 | Not detected (noun) | 腫瘍細胞不検出 |
| 23 | 検出されず | Not detected (negative passive) | 微小転移は検出されず |

*Notes: Patterns are applied to the character-level context window surrounding each extracted entity span in the deidentified analytic text. An entity span is classified as negated and excluded from the downstream analytic entity set if one or more of these patterns is detected in the surrounding context. Negation expressions themselves are typically not restored by the whitelist-based deidentification step and remain masked as asterisks in the deidentified analytic text. Examples are illustrative and drawn from synthetic clinical note fragments consistent with the analytic text format.*

**Table S4.** Incidence/Prevalence Classification and Time-Window Sensitivity Analysis for ATC Code L Records (Layer C, n = 3,689 Patients)

| **Incidence classification** | **N patients** | **% of Layer C** | **ATC-L within 30 days** | **ATC-L within 60 days** | **ATC-L within 90 days** |
| --- | --- | --- | --- | --- | --- |
| **By incidence classification** | | | | | |
| Incident | 1,556 | 42.2% | 14.8% | 21.0% | 24.3% |
| Prevalent | 2,016 | 54.6% | 0.4% | 1.5% | 3.1% |
| Unclassifiable | 117 | 3.2% | 11.1% | 17.1% | 20.5% |
| **Overall** | | | | | |
| All patients (Layer C) | 3,689 | 100% | 6.8% | 10.2% | 12.6% |

*Notes: Incident = first recorded cancer diagnosis ≥30 days after the hospital's data capture start date. Prevalent = first diagnosis date predating the data capture start date. Unclassifiable = first diagnosis date 0–29 days from data capture start. ATC-L within N days = proportion of patients with at least one ATC code L (antineoplastic and immunomodulating agents) record in the claims data within N days of their first cancer diagnosis date. Data source: DATuM IDEA database; observation period varies by hospital. Layer C: downstream analyzable text cohort (3,689 patients / 38,841 documents).*
