# Supplementary material for: Balancing Privacy Protection and Reproducible Entity Extraction in Real-World Data in the Development of a Framework for Deidentified Japanese Electronic Health Record Narratives Using BERT: Retrospective Observational Study
Source: JMIR Med Inform. 2026 Nov 6;14:e97411. doi: 10.2196/97411 (PMC13743013; doi:10.2196/97411)
Supplement: Supplementary file 2 [file medinform-v14-e97411-s002.docx]

**Supplementary Methods**

**Procedure Keyword List for Identifying Cancer-Directed Procedures in Japanese Claims**

To identify cancer-directed procedures as a proxy for curative-intent treatment, procedure-name strings recorded in Japanese claims data were searched using an OR-based keyword approach. A record was classified as procedure-positive if the procedure name contained at least one of the following Japanese keywords: 腫瘍 (tumor), 全摘 (total resection/excision), 切除 (resection/excision), 塞栓 (embolization), or ラジオ波 (radiofrequency ablation).

Keyword-based identification may capture heterogeneous interventions and may not perfectly distinguish curative-intent from non-curative procedures. This procedure proxy was therefore used only for exploratory concordance analyses in the downstream analyzable text cohort and should be interpreted as an observable-treatment proxy rather than as a definitive stage assignment method.

**Conceptual Workflow for Deidentification and Entity Extraction**

This workflow reflects the governance-constrained separation between upstream processing of raw source text within ICI and downstream analyses on deidentified analytic text within TOPPAN.

**Within ICI (with access to raw source text)**

**1. BERT-based entity extraction from raw source text**
BERT-based extraction is applied directly to the raw source text. Entities identified include both clinical entities (eg, TNM components and stage expressions) and PHI categories (eg, personal names, contact information, addresses, institution names, and patient identifiers).

**1a. Automated upstream quality assurance**
Extracted entity spans are checked for format validity (eg, TNM value-range constraints and stage token membership) and corpus-level completeness. Records with anomalous extraction patterns are flagged automatically.

**1b. Manual upstream quality assurance**
A sample of flagged documents and a random sample of unflagged documents undergo expert review within ICI to verify extraction accuracy before any outputs are released downstream.

**2. Whitelist dictionary consultation**
A whitelist dictionary of safe expressions is also consulted. The dictionary is content-biased: Japanese function words (eg, conjunctions and particles) have a low probability of being included, whereas Japanese adverbs and many English function words (eg, particles such as “up,” “on,” “down,” and “out”; adpositions such as “in,” “under,” “towards,” and “before”) may be restored when matched.

**3. Generation of deidentified analytic text**
First, every character in the raw source text is replaced with a single-byte asterisk to produce a fully masked baseline.

Then selectively restore:

(a) BERT-extracted entities not labeled as PHI; and

(b) whitelist-matched tokens.
All other characters remain masked.

**4. Output to TOPPAN**
The following outputs are transferred to TOPPAN under the certified governance scheme:
(i) the deidentified analytic text; and
(ii) the corresponding non-PHI entity outputs from the BERT extractor.

**Within TOPPAN (with access only to deidentified outputs)**

**5. Receipt of deidentified analytic text**
TOPPAN receives the deidentified analytic text and the corresponding non-PHI entity outputs.

**6. Downstream SQL-based extraction for proxy-based analyses**
For the proxy-based concordance analyses reported in this study, TNM components and stage tokens are identified from the deidentified analytic text using SQL-based extraction logic independent of the upstream BERT-derived entity outputs.

**7. Patient-level aggregation for concordance analyses**
Extracted outputs are aggregated at the patient level according to study rules and compared with structured-data treatment proxies for proxy-based concordance analyses.

**Closing note**

The internal entity-level evaluation against the gold-standard reference set was performed at the BERT extraction step within ICI (Step 1) on raw source text, before the deidentified analytic text was generated; it is reported in Methods > Model Development and Internal Reference Evaluation. The downstream steps executed within TOPPAN operate exclusively on the deidentified analytic text and the non-PHI entity outputs received from ICI. This pseudocode is provided to support conceptual reproducibility and does not disclose proprietary implementation details or governed source data.
