# Supplementary material for: Balancing Privacy Protection and Reproducible Entity Extraction in Real-World Data in the Development of a Framework for Deidentified Japanese Electronic Health Record Narratives Using BERT: Retrospective Observational Study
Source: JMIR Med Inform. 2026 Nov 6;14:e97411. doi: 10.2196/97411 (PMC13743013; doi:10.2196/97411)
Supplement: Supplementary file 3 [file medinform-v14-e97411-s003.docx]

**The RECORD statement – checklist of items, extended from the STROBE statement, that should be reported in observational studies using routinely collected health data.**

|  | **Item No.** | **STROBE items** | **Location in manuscript where items are reported** | **RECORD items** | **Location in manuscript where items are reported** |
| --- | --- | --- | --- | --- | --- |
| **Title and abstract** | | | | | |
|  | 1 | (a) Indicate the study’s design with a commonly used term in the title or the abstract (b) Provide in the abstract an informative and balanced summary of what was done and what was found | Title (study design stated as “Retrospective Observational Study”); Abstract — Background, Objective, Methods, Results, Conclusions | RECORD 1.1: The type of data used should be specified in the title or abstract. When possible, the name of the databases used should be included.  RECORD 1.2: If applicable, the geographic region and timeframe within which the study took place should be reported in the title or abstract.  RECORD 1.3: If linkage between databases was conducted for the study, this should be clearly stated in the title or abstract. | RECORD 1.1: Title (real-world data; deidentified Japanese electronic health record narratives); Abstract — Methods (DATuM IDEA database; retrospective observational study). RECORD 1.2: Abstract — Methods (two hospitals in Japan; January 1, 2019, through June 30, 2025). RECORD 1.3: Abstract — Methods (deterministic linkage performed within ICI prior to deidentification). |
| **Introduction** | | | | | |
| Background rationale | 2 | Explain the scientific background and rationale for the investigation being reported | Introduction — Missing Clinical Context in Pharmacoepidemiologic Studies; Challenge: Privacy-Utility Trade-Off in Sharing Clinical Narratives |  |  |
| Objectives | 3 | State specific objectives, including any prespecified hypotheses | Introduction — Objective and Significance: Generating Auditable Deidentified Analytic Text |  |  |
| **Methods** | | | | | |
| Study Design | 4 | Present key elements of study design early in the paper | Methods — Study Design, Setting, Data Sources, and Governance (retrospective observational study using the DATuM IDEA database); Abstract — Methods |  |  |
| Setting | 5 | Describe the setting, locations, and relevant dates, including periods of recruitment, exposure, follow-up, and data collection | Methods — Study Design, Setting, Data Sources, and Governance (two oncology care hospitals in Japan; January 1, 2019, through June 30, 2025) |  |  |
| Participants | 6 | *(a) Cohort study* - Give the eligibility criteria, and the sources and methods of selection of participants. Describe methods of follow-up  *Case-control study* - Give the eligibility criteria, and the sources and methods of case ascertainment and control selection. Give the rationale for the choice of cases and controls  *Cross-sectional study* - Give the eligibility criteria, and the sources and methods of selection of participants  *(b) Cohort study* - For matched studies, give matching criteria and number of exposed and unexposed  *Case-control study* - For matched studies, give matching criteria and the number of controls per case | Methods — Study Population (eligibility criteria); Methods — Sampling Strategy | RECORD 6.1: The methods of study population selection (such as codes or algorithms used to identify subjects) should be listed in detail. If this is not possible, an explanation should be provided.  RECORD 6.2: Any validation studies of the codes or algorithms used to select the population should be referenced. If validation was conducted for this study and not published elsewhere, detailed methods and results should be provided.  RECORD 6.3: If the study involved linkage of databases, consider use of a flow diagram or other graphical display to demonstrate the data linkage process, including the number of individuals with linked data at each stage. | RECORD 6.1: Methods — Study Population (ICD-10 codes C00-D48; four-criteria definition (1)-(4) for the downstream analyzable text cohort [Layer C]); Methods — Sampling Strategy. RECORD 6.2: Validation of population selection algorithms against an independent gold standard remains outside the scope of the present study and is planned as future work (see Discussion — Limitations and Future Directions, Eleventh). RECORD 6.3: Methods — Data Linkage; Results — Data Linkage (deterministic linkage within ICI prior to deidentification; structured-record linkage available for 4346/4383 patients [99.2%] in the linked text cohort [Layer B]). |
| Variables | 7 | Clearly define all outcomes, exposures, predictors, potential confounders, and effect modifiers. Give diagnostic criteria, if applicable. | Methods — NLP Model for Information Extraction; Methods — Definition of PHI; Methods — Conceptual Overview of the Deidentification and Extraction Pipeline; Methods — Evaluation Framework; Methods — Downstream Recoverability of TNM and Stage-Related Mentions; Methods — Internal Logical Consistency; Methods — Proxy-Based Concordance With Structured Data | RECORD 7.1: A complete list of codes and algorithms used to classify exposures, outcomes, confounders, and effect modifiers should be provided. If these cannot be reported, an explanation should be provided. | RECORD 7.1: Methods — NLP Model for Information Extraction; Methods — Negation Handling (23-pattern postextraction negation filter for excluding negated clinical entity mentions; Table S3 in Multimedia Appendix 1); Methods — Model Development and Internal Reference Evaluation; Methods — Conceptual Overview of the Deidentification and Extraction Pipeline; Multimedia Appendix 2 — Procedure Keyword List for Identifying Cancer-Directed Procedures in Japanese Claims; Multimedia Appendix 2 — Conceptual Workflow for Deidentification and Entity Extraction (stepwise pseudocode); Table S3 in Multimedia Appendix 1 (negation patterns). |
| Data sources/ measurement | 8 | For each variable of interest, give sources of data and details of methods of assessment (measurement).  Describe comparability of assessment methods if there is more than one group | Methods — Study Design, Setting, Data Sources, and Governance; Methods — Data Linkage; Methods — Missing and Unavailable Data (RECORD Compliance; Table S1 in Multimedia Appendix 1); Methods — NLP Model for Information Extraction; Methods — Model Development and Internal Reference Evaluation; Methods — Conceptual Overview of the Deidentification and Extraction Pipeline; Methods — Deidentification Metrics (Masking and Restoration Rates); Methods — Proxy-Based Concordance With Structured Data |  |  |
| Bias | 9 | Describe any efforts to address potential sources of bias | Methods — Sampling Strategy (conditional construction of the downstream analyzable text cohort [Layer C]); Methods — Evaluation Framework; Methods — Study Population (Layer C used for marginal recoverability and Layer D for conditional recoverability); Results — Study Population and Analytic Flow (Layer D comprising 36.3% of patients and 30.7% of documents in Layer C; this distinction characterizes the conditioning introduced by the recoverable subset definition); Discussion — Limitations and Future Directions (first: cohort conditioning; eighth: cross-hospital generalizability) |  |  |
| Study size | 10 | Explain how the study size was arrived at | Methods — Study Population (four-criteria analytic cohort definition); Results — Study Population and Analytic Flow (51,876 → 10,214 [Layer A] → 4383 [Layer B] → 3689 patients/38,841 documents [Layer C] → 1339 patients/11,914 documents with a recoverable TNM or stage mention [Layer D]); Figure 1 (four-layer cohort attrition diagram) |  |  |
| Quantitative variables | 11 | Explain how quantitative variables were handled in the analyses. If applicable, describe which groupings were chosen, and why | Methods — Deidentification Metrics (Masking and Restoration Rates) (text opacity rate; explicitly not a measure of readability, linguistic coherence, or privacy guarantee); Methods — Downstream Recoverability of TNM and Stage-Related Mentions (operational definition of marginal and conditional recoverability); Methods — Internal Logical Consistency (overall agreement, positive/negative percent agreement [PPA/NPA], Cohen kappa, and Jaccard coefficients); Methods — Proxy-Based Concordance With Structured Data; Methods — Observation Time Definition; Methods — Incident and Prevalent Case Classification and Time Window Sensitivity |  |  |
| Statistical methods | 12 | (a) Describe all statistical methods, including those used to control for confounding  (b) Describe any methods used to examine subgroups and interactions  (c) Explain how missing data were addressed  (d) *Cohort study* - If applicable, explain how loss to follow-up was addressed  *Case-control study* - If applicable, explain how matching of cases and controls was addressed  *Cross-sectional study* - If applicable, describe analytical methods taking account of sampling strategy  (e) Describe any sensitivity analyses | Methods — Evaluation Framework; Methods — Internal Logical Consistency; Methods — Proxy-Based Concordance With Structured Data; Methods — Manual Audit (Sanity Check) and Residual PHI Inspection; Methods — Missing and Unavailable Data (RECORD Compliance). Missing data at the variable level are addressed via RECORD-compliant variable availability analysis (Table S1 in Multimedia Appendix 1). A full gold-standard reference set evaluation with quantitative interannotator agreement (IAA F1=0.593, precision 0.579, recall 0.607) and Cohen kappa for chance-corrected concordance (κ=0.766) is reported in Methods — Model Development and Internal Reference Evaluation; Results — Internal Reference Evaluation of the Extraction Model; Table S2 in Multimedia Appendix 1. |  |  |
| Data access and cleaning methods |  | .. | .. | RECORD 12.1: Authors should describe the extent to which the investigators had access to the database population used to create the study population.  RECORD 12.2: Authors should provide information on the data cleaning methods used in the study. | RECORD 12.1: Methods — Data Access and Security (raw source text retained and processed within ICI; researchers at TOPPAN accessed only deidentified outputs and had no access to direct identifiers). RECORD 12.2: Methods — Model Development and Internal Reference Evaluation (text_tag_info standardization: tab characters replaced with 4 half-width spaces, and CR/LF/CRLF line breaks replaced with HTML break markers prior to annotation and model input). |
| Linkage |  | .. | .. | RECORD 12.3: State whether the study included person-level, institutional-level, or other data linkage across two or more databases. The methods of linkage and methods of linkage quality evaluation should be provided. | RECORD 12.3: Methods — Data Linkage (deterministic person-level linkage performed within ICI before deidentification at each participating hospital, using patient chart identifiers to link unstructured EHR narratives with structured records); Results — Data Linkage (structured-record linkage available for 4346/4383 patients [99.2%] in the linked text cohort [Layer B]). |
| **Results** | | | | | |
| Participants | 13 | (a) Report the numbers of individuals at each stage of the study (*e.g.*, numbers potentially eligible, examined for eligibility, confirmed eligible, included in the study, completing follow-up, and analysed)  (b) Give reasons for non-participation at each stage.  (c) Consider use of a flow diagram | Results — Study Population and Analytic Flow (51,876 to 10,214 [Layer A] to 4383 patients with linked progress notes [Layer B] to 3689 patients/38,841 documents in the downstream analyzable text cohort [Layer C], of whom 1339 patients/11,914 documents had a recoverable TNM or stage mention [Layer D]); Figure 1 (four-layer cohort attrition diagram) | RECORD 13.1: Describe in detail the selection of the persons included in the study (*i.e.,* study population selection) including filtering based on data quality, data availability and linkage. The selection of included persons can be described in the text and/or by means of the study flow diagram. | RECORD 13.1: Methods — Study Population (selection criteria); Methods — Sampling Strategy; Results — Study Population and Analytic Flow; Figure 1 (full cohort funnel showing each filtering step, including the Layer D subset defined by at least one recoverable TNM or stage mention). |
| Descriptive data | 14 | (a) Give characteristics of study participants (*e.g.*, demographic, clinical, social) and information on exposures and potential confounders  (b) Indicate the number of participants with missing data for each variable of interest  (c) *Cohort study* - summarise follow-up time (*e.g.*, average and total amount) | Results — Patient Characteristics and Observation Time in Major Cancer Subgroups; Table 2 (subgroup-specific patient and document characteristics in the downstream analyzable text cohort [Layer C]); Methods — Observation Time Definition; Methods — Missing and Unavailable Data (RECORD Compliance). A formal analysis of missing data at the variable level is reported: patient-level variables (age, sex, observation time) were fully available (0% unavailable); document-level extraction variables were structurally unavailable in 75.8% (T/N/M) to 76.4% (Stage) of documents; proxy-based concordance variables were unavailable in 70.3% (antineoplastic drug record) to 73.8% (procedure record) of patients (Table S1 in Multimedia Appendix 1). |  |  |
| Outcome data | 15 | *Cohort study* - Report numbers of outcome events or summary measures over time  *Case-control study* - Report numbers in each exposure category, or summary measures of exposure  *Cross-sectional study* - Report numbers of outcome events or summary measures | Results — Internal Reference Evaluation of the Extraction Model (entity-level recall, precision, F1 on gold-standard reference set; interannotator agreement F1=0.593, Cohen kappa=0.766; fine-tuned model F1=0.557 vs pretraining baseline F1=0.384; Table S2 in Multimedia Appendix 1); Results — Downstream Recoverability, Internal Logical Consistency, and Proxy-Based Concordance (Layer C marginal and Layer D conditional recoverability, internal logical consistency, and proxy-based concordance metrics); Table 3 (downstream recoverability, internal logical consistency, and proxy-based concordance metrics); Results — Deidentification Metrics by ICD-10 Category; Table 4 (masking/restoration rates); Methods — Incident and Prevalent Case Classification and Time Window Sensitivity; Table S4 in Multimedia Appendix 1; Results — Manual Audit (Sanity Check) and PHI Inspection |  |  |
| Main results | 16 | (a) Give unadjusted estimates and, if applicable, confounder-adjusted estimates and their precision (e.g., 95% confidence interval). Make clear which confounders were adjusted for and why they were included  (b) Report category boundaries when continuous variables were categorized  (c) If relevant, consider translating estimates of relative risk into absolute risk for a meaningful time period | Results — Internal Reference Evaluation of the Extraction Model (strict span-level interannotator agreement F1=0.593 and Cohen kappa=0.766; Table S2 in Multimedia Appendix 1); Results — Downstream Recoverability, Internal Logical Consistency, and Proxy-Based Concordance; Table 3; Results — Deidentification Metrics by ICD-10 Category; Table 4; Methods — Missing and Unavailable Data (RECORD Compliance) (variable-level availability; Table S1 in Multimedia Appendix 1). Cohen kappa was interpreted cautiously because of class imbalance, and Jaccard coefficients were reported as positive-set overlap measures. |  |  |
| Other analyses | 17 | Report other analyses done—e.g., analyses of subgroups and interactions, and sensitivity analyses | Results — Manual Audit (Sanity Check) and PHI Inspection (stratified manual audit of 400 deidentified narratives); Discussion — Manual Audit of Deidentified Outputs (2 independent auditors; approximately 30 overlapping documents). Cancer-type-stratified subgroup analyses are reported throughout Tables 2-4. Methods — Incident and Prevalent Case Classification and Time Window Sensitivity (time window sensitivity analyses for proxy-based concordance at 30, 60, and 90 days, stratified by incident [n=1556, 42.2%], prevalent [n=2016, 54.6%], and unclassifiable [n=117, 3.2%] status; Table S4 in Multimedia Appendix 1). |  |  |
| **Discussion** | | | | | |
| Key results | 18 | Summarise key results with reference to study objectives | Discussion — Principal Findings |  |  |
| Limitations | 19 | Discuss limitations of the study, taking into account sources of potential bias or imprecision. Discuss both direction and magnitude of any potential bias | Discussion — Limitations and Future Directions | RECORD 19.1: Discuss the implications of using data that were not created or collected to answer the specific research question(s). Include discussion of misclassification bias, unmeasured confounding, missing data, and changing eligibility over time, as they pertain to the study being reported. | RECORD 19.1: Discussion — Limitations and Future Directions, eleven numbered limitations: (1) conditional construction of the downstream analyzable text cohort [Layer C], addressed via marginal recoverability reporting (Layer D: 36.3% patient-level, 30.7% document-level); (2) annotation corpus length restriction (Q1–Q3); (3) exclusion of rare-cancer ICD-10 subgroups (<50 patients); (4) small NLP annotation corpus size, contextualized against IAA F1=0.593; (5) ATC code L Stage IV proxy including neoadjuvant and adjuvant therapies, and low PPV (0.031) of the early-stage procedure proxy; (6) short observation times constraining proxy-based concordance; (7) text opacity rate is not a privacy guarantee; (8) two-hospital generalizability not yet established; (9) proprietary code and rule inventory non-releasable; (10) detailed corpus description statistics not computed in this study because of ICI-side processing constraints; (11) population selection algorithms not independently validated against a patient-level gold standard. Discussion — Interpretation of Proxy-Based Concordance: Low PPV for Early-Stage Disease. |
| Interpretation | 20 | Give a cautious overall interpretation of results considering objectives, limitations, multiplicity of analyses, results from similar studies, and other relevant evidence | Discussion — Principal Findings; Discussion — Balancing Privacy Protection and Analytic Utility; Discussion — Extraction/Structuring Quality and Error Characteristics; Discussion — Interpretation of Proxy-Based Concordance: Low PPV for Early-Stage Disease; Discussion — Manual Audit of Deidentified Outputs |  |  |
| Generalisability | 21 | Discuss the generalisability (external validity) of the study results | Discussion — Limitations and Future Directions (data from two hospitals; generalizability across institutions and documentation styles; scaling considerations to 50 or more sites) |  |  |
| **Other Information** | | | | | |
| Funding | 22 | Give the source of funding and the role of the funders for the present study and, if applicable, for the original study on which the present article is based | Funding (No external funding was received) |  |  |
| Accessibility of protocol, raw data, and programming code |  | .. | .. | RECORD 22.1: Authors should provide information on how to access any supplemental information such as the study protocol, raw data, or programming code. | RECORD 22.1: Data Availability (DATuM IDEA database access governance; analytic code and selected implementation materials available upon reasonable request); Author note (patent application and proprietary boundaries); Multimedia Appendix 2 — Procedure Keyword List for Identifying Cancer-Directed Procedures in Japanese Claims; Multimedia Appendix 2 — Conceptual Workflow for Deidentification and Entity Extraction (stepwise pseudocode). |

*Reference: Benchimol EI, Smeeth L, Guttmann A, Harron K, Moher D, Petersen I, Sørensen HT, von Elm E, Langan SM, the RECORD Working Committee. The REporting of studies Conducted using Observational Routinely-collected health Data (RECORD) Statement. *PLoS Medicine* 2015; in press.

*Checklist is protected under Creative Commons Attribution ([CC BY](http://creativecommons.org/licenses/by/4.0/)) license.
